# Fluoroquinolone-Resistant Typhoid, South Africa

**DOI:** 10.3201/eid1605.091917

**Published:** 2010-05

**Authors:** Karen H. Keddy, Anthony M. Smith, Arvinda Sooka, Husna Ismail, Stephen Oliver

**Affiliations:** National Institute of Communicable Diseases, Johannesburg, South Africa (K.H. Keddy, A.M. Smith, A. Sooka, H. Ismail); University of the Witwatersrand, Johannesburg (K.H. Keddy, A.M. Smith, H. Ismail); National Health Laboratory Service, Groote Schuur, Cape Town, South Africa (S. Oliver); University of Cape Town, Cape Town (S. Oliver)

**Keywords:** Bacteria, Salmonella enterica Typhi, fluoroquinolone resistance, qnr genes, typhoid, South Africa, letter

**To the Editor**: *Salmonella enterica* serotype Typhi, the causal pathogen for typhoid, is a major public health hazard in many parts of the world, with an estimated 21.6 million cases of typhoid and 217,000 deaths occurring each year (*1*). Most isolates in South Africa are susceptible to quinolones, and fluoroquinolones remain the treatment of choice (*2*). The disease is primarily water or foodborne, but person-to-person spread is well recognized (*3*). Travelers to disease-endemic regions may be at risk for typhoid, which may result in the importation of strains of *S.* Typhi with unfamiliar or unusual resistance patterns (*4*). Such infections present a challenge to local clinicians on optimal patient management.

*S.* Typhi was isolated from the blood culture of a woman 65 years of age from Cape Town; she had been in contact with a traveler to Bangladesh. The patient was treated first with ciprofloxacin, but this medication was changed to high-dose ceftriaxone combined with doxycycline for 8 days; she recovered well. Contact tracing indicated no family members had typhoid fever or carried the organism. The person who had traveled to Bangladesh was unavailable to provide further history or a stool specimen. No other potential source of infection could be elucidated: the patient lived in an urban area with safe water sources and shared meals with her family.

The isolate was referred to the Enteric Diseases Reference Unit for confirmation of identification, serotyping (Kauffman-White scheme), and antimicrobial drug susceptibility testing using the Etest (bioMérieux, Marcy l’Étoile, France) and agar dilution methods, according to criteria of the Clinical and Laboratory Standards Institute (Wayne, PA, USA) (www.clsi.org). The isolate was resistant to ampicillin, chloramphenicol, sulfamethoxazole, nalidixic acid, and ciprofloxacin, but susceptible to ceftriaxone and tetracycline.

Pulsed-field gel electrophoresis (PFGE) analysis was performed on the isolate, following the standard PulseNet protocol (*5*). The PFGE pattern was compared with a database of *S.* Typhi PFGE patterns from South Africa by using BioNumerics version 6.01 software (Applied Maths, Sint-Martens-Latem, Belgium). The PFGE pattern of this isolate was 100% identical to pattern JPPX01.0026 in the Global PulseNet *Salmonella* Typhi Database. This pattern, which has been reported to PulseNet from India, Kenya, Tanzania, and Taiwan, is the most common pattern in the database (www.pulsenetinternational.org/projects/styphidatabase.asp); it is rarely seen in South Africa (Figure).

**Figure:**
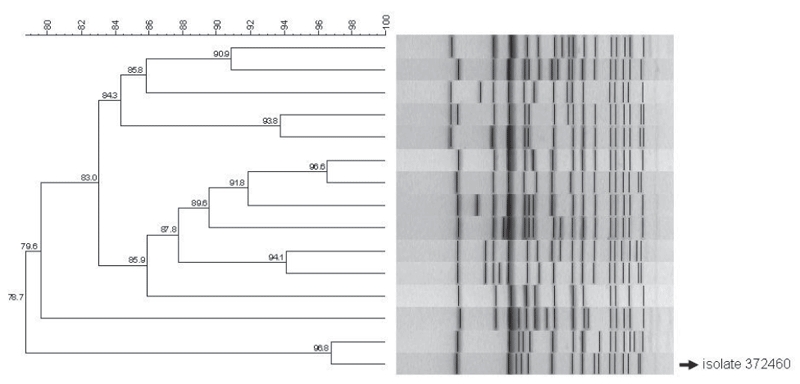
Dendrogram of pulsed-field gel electrophoresis patterns representative of the 15 largest clusters of *Salmonella* *enterica* serotype Typhi isolates identified in South Africa during 2005–2009. The pattern of isolate 372460 is indicated. Scale bar represents percentage similarity of pathogens.

PCR was used to isolate the quinolone resistance-determining region (QRDR) of *gyrA*, *gyrB*, *parC,* and *parE* (*6*). Genes were sequenced by using the BigDye Terminator Cycle Sequencing Kit (Applied Biosystems, Foster City, CA, USA) and an Applied Biosystems 3130 genetic analyzer. The QRDR DNA sequences were compared with those of *S.* Typhi strain Ty2 (GenBank accession no. AE014613). PCR also used to confirm the presence of *qnrA*, *qnrB,* and *qnrS* genes (*6*). Analysis for mutations in the QRDR of *gyrA*, *gyrB*, *parC,* and *par* found a single amino-acid mutation (Ser83 to Tyr) in g*yrA*. No amino acid mutations were identified in g*yrB*, *parC*, and *parE*. PCR for detection of *qnr* genes confirmed the presence of a *qnrS* gene, which was identified as the *qnrS1* variant by nucleotide sequence analysis.

The efflux of quinolones from bacterial cells was investigated in the following manner. For nalidixic acid and ciprofloxacin, agar dilution MIC testing was performed in the absence and presence of 40 µg/mL of the efflux pump inhibitor, Phe-Arg-β-naphthylamide (*6*). In the presence of efflux pump inhibitor, the MIC to ciprofloxacin decreased from 4 µg/mL to 1 µg/mL, and the MIC to nalidixic acid decreased from >512 µg/mL to 32 µg/mL, establishing the involvement of an efflux pump in conferring quinolone resistance.

The mutation in g*yrA* (Ser83 to Tyr) confers reduced susceptibility to ciprofloxacin to a maximum MIC ≈0.5 µg/mL (*7*) and the QnrS1 protein confers reduced susceptibility to ciprofloxacin to a maximum MIC ≈0.5 µg/mL (*8*). We showed that a single amino acid mutation in *gyrA* (Ser83 to Tyr) with the QnrS1 protein and active efflux, conferred ciprofloxacin resistance, at least to an MIC level of 4 µg/mL. Previously, Smith et al. reported quinolone resistance in South African isolates of *S.* Typhi mediated by mutations in *gyrA* and *parC* in combination with active efflux (*6*). We report *qnrS1* from *S.* Typhi, confirming the role of plasmid-mediated fluoroquinolone resistance in *S.* Typhi (*9*) and a fluoroquinolone- resistant strain in South Africa. Fluoroquinolone resistance is well recognized in Bangladesh; other researchers have described multidrug-resistant *S.* Typhi isolates imported from that country (*10*). Molecular epidemiology supports the conclusion that this strain likely originated in Bangladesh (L. Theobald, pers.comm.).

In conclusion, fluoroquinolone-resistant typhoid fever is a reality in South Africa in patients who have a history of travel or contact with travelers. Blood cultures are mandatory to guide antimicrobial drug management. Plasmid-mediated fluoroquinolone resistance has implications for cotransference of resistance to the major antimicrobial agents used to treat typhoid fever and for the potential for rapid spread of fluoroquinolone resistance through *S.* Typhi strains in South Africa. The presence of fluoroquinolone-resistant typhoid fever could force a change in current treatment guidelines for this disease.
